# Complete mitochondrial genome sequences from five *Eimeria* species (Apicomplexa; Coccidia; Eimeriidae) infecting domestic turkeys

**DOI:** 10.1186/1756-3305-7-335

**Published:** 2014-07-17

**Authors:** Mosun E Ogedengbe, Shiem El-Sherry, Julia Whale, John R Barta

**Affiliations:** 1 Department of Pathobiology, Ontario Veterinary College, University of Guelph, Guelph, ON N1G 2W1, Canada; 2 Department of Poultry Diseases, Faculty of Veterinary Medicine, Assiut University, Assiut 71526, Egypt

**Keywords:** Poultry coccidiosis, Evolution, Mitochondrial DNA, Parasitology, Diagnosis

## Abstract

**Background:**

Clinical and subclinical coccidiosis is cosmopolitan and inflicts significant losses to the poultry industry globally. Seven named *Eimeria* species are responsible for coccidiosis in turkeys: *Eimeria dispersa*; *Eimeria meleagrimitis*; *Eimeria gallopavonis*; *Eimeria meleagridis*; *Eimeria adenoeides*; *Eimeria innocua*; and, *Eimeria subrotunda*. Although attempts have been made to characterize these parasites molecularly at the nuclear 18S rDNA and ITS loci, the maternally-derived and mitotically replicating mitochondrial genome may be more suited for species level molecular work; however, only limited sequence data are available for *Eimeria* spp. infecting turkeys. The purpose of this study was to sequence and annotate the complete mitochondrial genomes from 5 *Eimeria* species that commonly infect the domestic turkey (*Meleagris gallopavo*).

**Methods:**

Six single-oocyst derived cultures of five *Eimeria* species infecting turkeys were PCR-amplified and sequenced completely prior to detailed annotation. Resulting sequences were aligned and used in phylogenetic analyses (BI, ML, and MP) that included complete mitochondrial genomes from 16 *Eimeria* species or concatenated CDS sequences from each genome.

**Results:**

Complete mitochondrial genome sequences were obtained for *Eimeria adenoeides* Guelph, 6211 bp; *Eimeria dispersa* Briston, 6238 bp; *Eimeria meleagridis* USAR97-01, 6212 bp; *Eimeria meleagrimitis* USMN08-01, 6165 bp; *Eimeria gallopavonis* Weybridge, 6215 bp; and *Eimeria gallopavonis* USKS06-01, 6215 bp). The order, orientation and CDS lengths of the three protein coding genes (COI, COIII and CytB) as well as rDNA fragments encoding ribosomal large and small subunit rRNA were conserved among all sequences. Pairwise sequence identities between species ranged from 88.1% to 98.2%; sequence variability was concentrated within CDS or between rDNA fragments (where indels were common). No phylogenetic reconstruction supported monophyly of *Eimeria* species infecting turkeys; *Eimeria dispersa* may have arisen via host switching from another avian host. Phylogenetic analyses suggest *E. necatrix* and *E. tenella* are related distantly to other *Eimeria* of chickens.

**Conclusions:**

Mitochondrial genomes of *Eimeria* species sequenced to date are highly conserved with regard to gene content and structure. Nonetheless, complete mitochondrial genome sequences and, particularly the three CDS, possess sufficient sequence variability for differentiating *Eimeria* species of poultry. The mitochondrial genome sequences are highly suited for molecular diagnostics and phylogenetics of coccidia and, potentially, genetic markers for molecular epidemiology.

## Background

As many as seven *Eimeria* species, *Eimeria dispersa, Eimeria meleagrimitis*, *Eimeria gallopavonis*, *Eimeria meleagridis*, *Eimeria adenoeides*, *Eimeria innocua* and *Eimeria subrotunda*, can cause coccidiosis in the turkey, *Meleagris gallopavo*[1]. Coccidiosis is widespread and pathogenic with considerable economic losses to the poultry industry [2,3]. These parasites possess morphotypes of oocysts with overlapping biological features that make identification, characterization and diagnosis challenging [4,2]. Delimiting individual species using morphological features, even when supplemented by 18S rDNA or internal transcribed spacer (ITS) sequence data, has been reported to be less than ideal for coccidia, especially for closely related parasites [5-10]. Sequences from the mitochondrial cytochrome *c* oxidase subunit I gene (mtCOI) have been shown to be reliable for delimiting closely related species [9] and the mtCOI locus appears to lack paralog issues associated with rDNA of these parasites [10].

A single, complete mitochondrial (mt) genome copy for parasites within the Apicomplexa is about 6 KB long [11,12]. Genome organisation varies considerably among eukaryotes in general and also within the Apicomplexa [13,14]. Among apicomplexan parasites, genome structures that have been reported include linear concatemers [15,16], linear genomes with terminal inverted telomeric repeats [17,18] and circular genomes [12,19]. Regardless of overall genome structure, all apicomplexan mt genomes examined to date possess three genes encoding cytochrome *c* oxidase subunit I (COI), cytochrome *c* oxidase subunit III (COIII) and cytochrome b (CytB), as well as numerous fragments of discontinuous and scrambled small subunits (SSU) and large subunit (LSU) rDNA. The specific LSU and SSU rDNA fragments found in the mt genome of apicomplexan parasites differ among distantly related parasites. Unlike many eukaryotic mt genomes, apicomplexan mt genomes do not encode 5S rRNA or tRNAs [20-24].

In the present study we report six new PCR generated, complete mt genome sequences from single oocyst derived lines of five *Eimeria* species infecting turkeys: *Eimeria dispersa* Briston strain, *Eimeria meleagrimitis* USMN08-01 strain, *Eimeria gallopavonis* Weybridge strain, *Eimeria gallopavonis* USKS06-01 strain, *Eimeria meleagridis* USAR97-01 strain and *Eimeria adenoeides* Guelph strain.

## Methods

### Parasites

Six single oocyst-derived lines of five *Eimeria* species were used in this study. A description of the origins of the original isolates from which each line was derived is provided by El Sherry *et al.*[25]. The resulting lines used were as follows: 1) *Eimeria adenoeides* Guelph strain [26] in submission, for biological features of the line), 2) *Eimeria dispersa* Briston strain; 3) *E. meleagrimitis* USMN08-01 strain see [27,28] for biological features); 4) *E. meleagridis* USAR97-01 strain see [29], for biological features); 5) *E. gallopavonis* Weybridge strain see [30] for biological features); and 6) *E. gallopavonis* USKS06-01 strain. All lines were derived from parent isolates using the method of Remmler and McGregor [31] with the modification that agar plugs carrying a single oocyst were given within gelatin capsules orally to specific-parasite free poults. All animal experimentation was conducted in SPF birds at the Campus Animal Facility, (University of Guelph, Guelph ON, Canada); all experimental procedures were reviewed and approved by the University of Guelph’s Animal Care Committee and complied with the Canadian Council on Animal Care’s Guide to the Care and Use of Experimental Animals (2nd edition).

### DNA extraction and long PCR amplification

Purification of oocysts and genomic DNA extraction was carried out as previously described by Ogedengbe *et al.*[9,24]. Mitochondrial whole genome amplification for all five *Eimeria* species was initiated using two sets of specific primers that generated overlapping PCR fragments: 1) Cocci_MT-WG-F (5′-TACACCTAGCCAACACGAT-3′) and Cocci_MT-WG-R (5′-GCAGCTGTAGATGGATGCTT-3′); and, 2) Inv_COI_262R (5′-AAWGCGGCATCRTAGAATTG-3′) and Inv_COI_461F (5′-CTAGCYATGGGATGTATTACTG-3′). Primers were designed from highly conserved regions within publically available mitochondrial genome sequences for *Eimeria* species infecting chickens (see Ogedengbe *et al*. 2013 for the species used in the primer design). The primer pairs Inv_COI_461F and Inv_COI_262R annealed 148 bp apart at bp 2069-2090 and bp 1920-1901 respectively, and the primer pairs Cocci_MT-WG-F and Cocci_MT-WG-R annealed 97 bp apart at bp 6322-6340 and bp 6224-6205, respectively, on the published mitochondrial genome sequence of *Eimeria mitis* [GenBank: KF501573]. Each pair of primers was used independently in a 50 μl reaction. PCR reactions using QIAGEN LongRange PCR kit (QIAGEN, Valencia, CA, USA) protocol according to the manufacturer’s instructions with the modification that an additional 1.5 mM MgCl_2_ was added to the PCR buffer provided by the manufacturer. For each *Eimeria* species, long PCR reactions consisted of ~200 ng genomic DNA template (when using the Inv_COI_461F/Inv_COI_262R primers), and 25 ng genomic DNA template (when using primers Cocci_MT-WG-F and Cocci_MT-WG-R), 1× LongRange PCR buffer, 4 mM MgCl_2_, 500 μM of each dNTP, 2U LongRange PCR enzyme mix and 0.4 μM of each primer. The PCR reaction profile consisted of denaturing at 93°C for 3 min followed by 35 cycles of 93°C for 15 s, 50°C for 30 s, 68°C for 6 min with a final extension cycle of 68°C for 10 min in an MJ mini thermal cycler (Bio Rad, CA, USA). PCR products were electrophoresed at 50 V through a 0.8% agarose gel prepared with 1 × TAE buffer containing ethidium bromide. DNA bands were viewed using UV transillumination (Spectronics Corporation, New York, USA) and their sizes were compared to a 100 bp to 10 kb DNA ladder (Bio Basic Inc., Mississauga ON, Canada). DNA bands were excised from the gel and purified using a QIA quick gel extraction and purification kit (Qiagen, Toronto ON, Canada) according to the manufacturer’s instruction.

### Sequencing

Purified PCR products were sequenced in both directions using a primer-walking strategy to generate near–complete mitochondrial genomes essentially as described by Ogedengbe *et al*. [24]. Sequencing was carried out using the ABI PRISM 7000 Sequence Detection System (Applied Biosystem Inc., Foster City, CA, USA) at the Laboratory services Division, University of Guelph (Guelph, ON, Canada).

### Sequence data assembly and analysis

The de novo sequence assembler within Geneious bioinformatics software (Version 6.1 and later versions, available from http://www.geneious.com) was used to trim and assemble Sanger sequencing chromatograms into high quality contigs for the primary PCR product from each species. To complete each mt genome, PCR products were generated using a reverse primer downstream of the original forward primer (i.e. Cocci_MT-WG-F or Inv_COI_461F) and a forward primer upstream of the original reverse primer (i.e. Cocci_MT-WG-R or Inv_COI_262R, respectively); primers were designed such that a minimum of 100 bp of the resulting fragment overlapped the original long PCR product at each end. Each resulting PCR product was sequenced in both directions and the resulting consensus sequence was used to fill in the region between the two original long PCR amplification primers. The coding genes and rDNA fragments were first mapped by comparison with other *Eimeria* mt genomes (i.e. *E. tenella* AB564272, annotated by Hikosaka et al. [22], and *E. mitis* KF501573, annotated by Ogedengbe *et al.*[24]. Additional putative rDNA fragments were identified by comparing well conserved unannotated regions found in all of the aligned *Eimeria* sp. genomes to the mt genome of *Plasmodium falciparum* (M76611). Sequence identity between such conserved regions and rDNA fragments from *P. falciparum* greater than 60% were mapped as putative rDNA fragments. Putative start and stop codon positions for each of the coding DNA sequences (CDS) were identified following methods previously described by Ogedengbe *et al*. [24]. Translations using the mold/protozoan mitochondrial codon translation (i.e. translation_table_4) were searched using Blastp against the non redundant sequence database to confim the identity of the translation product produced by each CDS. Base compositions and nucleotide changes within the CDS among the six mt genome sequences were analysed from within the Geneious software package.

### Phylogenetic analyses

The six newly generated, PCR–based mt genome sequences of *Eimeria* spp infecting turkey: *Eimeria dispersa* Briston strain; *E. meleagrimitis* USMN08-01 strain; *E. meleagridis* USAR97-01 strain; *E. adenoeides* Guelph strain and *E. gallopavonis* Weybridge strain; *E. gallopavonis* USKS06-01 strain were aligned with the 10 publically available complete mt genome sequences from seven *Eimeria* spp. infecting chickens and *Eimeria magna* that infects rabbits (i.e. all available apicomplexan taxa that had the same genome structure). Three sequences of *Eimeria mitis* (KCE409029; KC409030 and KC409031) generated from clones were not included in the phylogenetic analysis because of the likelihood of PCR artifacts in these sequences as documented by Ogedengbe *et al.*[24]. GenBank sequence accession numbers are indicated on the trees.

To permit whole genome alignments, all mt genome sequences were linearized at the same position, 85-87 nt upstream of the small subunit rDNA fragment SSU/A corresponding to the binding site of the Cocci_MT-WG-F primer. Linearized sequences were aligned based on the primary structure using the multiple sequence alignment algorithm implemented from within Geneious 6.1; indels downstream of the Cocci_MT-WG-R primer binding site made unambiguous alignment in that region unlikely so the short sequences downstream of this primer binding region (44 to 97 bp depending on the *Eimeria* sp.) were not included in subsequent phylogenetic analyses using whole genome sequences.

Regions between rDNA fragments contained frequent indels that made unambiguous alignment of these regions difficult and the CDS for the three genes contained the majority of the genetic diversity found within the mt genomes. For these reasons, we chose to use concatenated CDS for CytB, COI and COIII (or their corresponding amino acid sequences) as datasets for phylogenetic analyses. The sequence data was thus partitioned into 3 datasets as follow: 1) a global nucleotide sequence data set for all 16 whole genome sequences (after removal of the short regions downstream of the Cocci_MT-WG-R primer); 2) concatenated DNA sequences for the 3 CDS; 3) concatenated amino acid (aa) translations of the 3 CDS.

Phylogenetic analyses were performed on all 3 data sets using three tree building methods, Bayesian analysis (BI) [32] performed using MrBayes (Version 3.2.), Maximum Parsimony (MP) using PAUP 4.0 [33] and Maximum Likelihood (ML) using PhyML [34], executed from within the Geneious bioinformatics software package (Version 6.1 and later versions).

Data set one, consisting of whole mt genome nucleotide sequences (excluding the short regions downstream of the Cocci_MT-WG-R primer), for all 16 whole genome sequences and data set two, consisting of concatenated CDS were analysed using all three tree-building methods (BI, ML or MP). Selection of the best fit evolutionary model for the BI and ML analyses was evaluated in both MrModeltest v2.3 (Nylander J. A. A. 2004. MrModeltest v2. Program; distributed by the author, Evolutionary Biology Centre, Uppsala University) and MEGA [35]. For the Bayesian analyses, Markov Chain Monte Carlo was performed for 1,000,000 generations with four chains and heated chain temperature of 0.2. The burn-in length was set at 400,000 and subsample frequency of 1000 [32,36]. For the ML analyses, 500 bootstrap replicates were calculated to estimate node support. In the MP analyses, characters were unordered and given equal weight; trees were searched using the branch and bound search algorithm.

Data set three, consisting of concatenated amino acid (aa) translations of the 3 CDS was analysed with the same three tree-building methods. The empirical Jones-Taylor-Thornton (JTT) model of amino acid substitution with gamma distribution frequency (G + F) for all sites by Jones *et al.*[37] was selected for the ML and BI analyses. Substitution models were assessed in MEGA [35]. Where outgroup rooting was appropriate, the *Eimeria magna* mitochondrial genome sequence [GenBank: KF419217] was used as the functional outgroup.

Although sequences were truncated to remove the short indel-rich region downstream of the reverse primer for the phylogenetic analyses, complete genome sequence alignments were used within Geneious for calculating the pairwise genetic distances and number of nucleotide differences among the six newly sequenced genome sequences.

## Results

### Six mt genomes from five *Eimeria* species infecting turkeys

The six complete mitochondrial genome sequences obtained from direct sequencing of PCR products from five *Eimeria* spp infecting turkeys varied modestly in their lengths: *Eimeria adenoeides* Guelph strain [GenBank: KJ608415, 6211 bp]; *Eimeria dispersa* Briston strain [GenBank: KJ608416, 6238 bp]; *Eimeria meleagridis* USAR97-01 strain [GenBank: KJ608418, 6212 bp]; *Eimeria meleagrimitis* USMN08-01 strain [GenBank: KJ608414, 6165 bp]; *Eimeria gallopavonis* Weybridge strain [GenBank: KJ608413, 6215 bp]; and, *Eimeria gallopavonis* USKS06-01 strain [GenBank: KJ608417, 6215 bp]. Base composition was A/T biased in all species (Table 1). Pairwise comparisons of the 6 aligned mt genome sequences from *Eimeria* spp. infecting turkeys (Table 2) indicate a high degree of sequence identity among these new genome sequences; 5311 nucleotide positions (84.9% of the aligned sequence lengths) were invariant among all 6 genome sequences. There was no intraspecific variation noted between two strains of *E. gallopavonis* (Weybridge and USKS06-01) whose complete mt genomes were identical. The two closely related *Eimeria* spp. causing ‘cecal coccidiosis’ in turkeys (i.e. *E. adenoeides* and *E. meleagridis*) demonstrated a genetic distance of 1.8% from each other and each was 3.1% divergent from the two strains of *E. gallopavonis* (Table 2). The physical form of the mitochondrial genomes was not directly assessed in this study, however, the mt genomes of these *Eimeria* species must be either linear concatenated or circular to permit successful PCR amplification of near full length mt genomes.

**Table 1:** **Base composition of whole mitochondrial genomes from five *Eimeria *** **species infecting turkeys**

**Parasite**	**A/T (%)**	**Adenine (%)**	**Cytosine (%)**	**Guanine (%)**	**Thymine (%)**
*Eimeria adenoeides* Guelph [GenBank: KJ608415]	64.6	29.8	18.4	17.0	34.8
*Eimeria dispersa* Briston [GenBank: KJ608416]	65.5	29.9	17.6	16.8	35.6
*Eimeria gallopavonis* USKS06-01 [GenBank: KJ608417]	64.9	30.1	18.3	16.9	34.8
*Eimeria gallopavonis* Weybridge [GenBank: KJ608413]	64.9	30.1	18.3	16.9	34.8
*Eimeria meleagridis* USAR97-01 [GenBank: KJ608418]	64.7	29.8	18.3	17.0	34.9
*Eimeria meleagrimitis* USMN08-01 [GenBank: KJ608414]	63.5	30.0	19.2	17.3	36.5

**Table 2:** **Percent pairwise sequence identities (lower values) and number of nucleotide differences (upper values) for six mitochondrial genomes from five *Eimeria *** **species that infect turkeys**

	***Eimeria dispersa *** **Briston [GenBank: KJ608416]**	***Eimeria meleagrimitis *** **USMN08-01 [GenBank: KJ608414]**	***Eimeria adenoeides *** **Guelph [GenBank: KJ608415]**	***Eimeria meleagridis *** **USAR97-01 [GenBank: KJ608418]**	***Eimeria gallopavonis *** **Weybridge [GenBank: KJ608413]**	***Eimeria gallopavonis *** **USKS06-01 [GenBank: KJ608417]**
***Eimeria dispersa *** **Briston**		742	472	486	486	486
[GenBank: KJ608416]						
***Eimeria meleagrimitis *** **USMN08-01**	88.1%		608	597	602	602
[GenBank: KJ608414]						
***Eimeria adenoeides *** **Guelph**	92.4%	90.2%		113	194	194
[GenBank: KJ608415 ]						
***Eimeria meleagridis *** **USAR97-01**	92.2%	90.4%	98.2%		191	191
[GenBank: KJ608418]						
***Eimeria gallopavonis *** **Weybridge**	92.2%	90.3%	96.9%	96.9%		0
[GenBank: KJ608413]						
***Eimeria gallopavonis *** **USKS06-01**	92.2%	90.3%	96.9%	96.9%	100%	
[GenBank: KJ608417]						

### Genome organization

**Figure 1:**
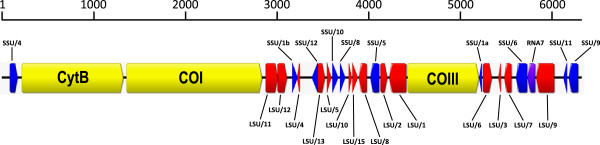
**Mitochondrial genome organization of five *Eimeria *species (Apicomplexa; Eimeriidae) infecting turkeys, *Meleagris gallopavo *(Aves; Galliformes).** Transcriptional direction and order of the three coding regions for CytB, COI and COIII (direction indicated by arrowed end) are identical to those of other *Eimeria* species. Fragments of LSU rDNA (lower LSU labels), SSU rDNA fragments (upper SSU labels) and unassigned RNA7 (lower label) were found in regions outside of the protein-coding genes. Naming of ribosomal DNA fragments follows the convention of Feagin *et al.* (2012, Table 1).

Genome content and organisation of all six *Eimeria* sp. mt genomes consisted of three protein-coding genes (COI, COIII and CytB) interspersed with 15 LSU and 11 SSU rDNA fragments (Figure 1). Pairwise sequence alignments between individual rDNA regions annotated in the *Eimeria* sp. genomes and the corresponding rDNA fragments of *P. falciparum* (M76611) identified by Feagin *et al.*[19] demonstrated pairwise sequence identities that ranged from 68.5% to 93.8%.

Searching conserved regions along the aligned mt genomes in the present study against the annotated *P. falciparum* mt genome identified three additional regions that are putative rDNA. The first two regions had high sequence identity to a single rDNA of *P. falciparum* encoding RNA14 (SSU/1) that appears to have been further fragmented on the *Eimeria* sp. mt genomes; the two resulting smaller fragments were found to map to two widely separated regions on these genomes. The first 29 bp of RNA14 from the *P. falciparum* mt genome (bp 5576-5548 of M76611) has high pairwise sequence identity (~79%) to a region designated RNA14a on the mt genomes of all *Eimeria* spp. The following 41 bp of RNA14 from the *P. falciparum* mt genome (bp 5547–5508 of M76611) has high pairwise sequence identity (75.6%) to a region designated RNA14b on the six mt genomes reported in the present study. The newly annotated rDNA fragment RNA14a (SSU/1a) was found in reverse orientation starting at bp 5141-5110 (varies in each *Eimeria* sp. mt genome) and RNA14b (SSU/1b) was found in forward orientation starting at bp 3104–3111 (varies in each *Eimeria* sp. mt genome). The remaining conserved region for which high sequence identity was discovered with the mt genome of *P. falciparum* corresponded to RNA5 (SSU/9) annotated by Feagin *et al*. [19]. This putative rDNA fragment was found in reverse orientation starting at bp 6130-6199 (varies in each *Eimeria* sp. mt genome) and corresponded to bp 4724-4802 on the *P. falciparum* mt genome. Although the pairwise sequence identity between the complete RNA5 (SSU/9) regions on the *Eimeria* spp. and *P. falciparum* mt genomes was only 63.2%, both the 5′ and 3′ ends of these regions were highly conserved (i.e. 80-85% sequence identity in the 20 bp at each end of the region).

The COIII CDS was most divergent (76.3% identical sites across the six mt genome sequences). The COI and CytB CDS showed 81.2% and 81.8% identical sites, respectively. Of the 272 sites demonstrating variation among the 6 COI CDS examined, 239 were synonymous (K_S_) changes and 33 were non-synonymous (K_A_) changes. The COIII CDS had 179 sites with variation (74 K_S_ and 40 K_A_) and the CytB CDS had 197 variable sites (167 K_S_ and 30 K_A_ changes). The CDS were more divergent than the rDNA fragments (80.2% sequence identity over the 3279 bp of the genomes identified as CDS versus 95.9% sequence identity over the 1880 bp identified as rDNA regions). Nucleotide differences and indels were observed within some fragmented rDNA regions but were most commonly observed within intergenic regions (i.e. between regions annotated as CDS or rDNA).

### Start codon determinations for COI, COIII and CytB

Start codon assignments were made by comparison with 13 publically available complete mt genomes from various *Eimeria* species and subsequent confirmation of appropriate open-reading frames. In the six mt genomes obtained in this study, an ATG start codon for the CytB CDS beginning 214 or 215 bp downstream of the start of the Cocci_MT-WG-F primer binding site was shared among all *Eimeria* spp. for which complete mitochondrial sequences have been obtained. Preceding the ATG start codon was a poly–T-rich ‘GTTTATGTTTA’ motif that was conserved in all *Eimeria* spp. of turkeys with the exception of *Eimeria meleagrimitis* USMN08-01. The latter sequence had a single substitution of ‘T’ with a ‘C’ producing a slightly different motif ‘GTTTATGTTCA’. A single stop codon, TAA, terminated the CDS for CytB, COI and COIII in all six mt genome sequences. Potential start codons for the COI CDS identified upstream of the highly conserved ‘Asn–His–Lys’ motif associated with the start of the heme–copper oxidase subunit I core region of COI were numerous for most of the new genome sequences except for *E. meleagrimitis* that had only 2 ORF’s that start upstream of that functionally conserved region c.f. [24]. Of these two potential start codons for COI in the *E. meleagrimitis* sequence, only one potential start codon was shared among all *Eimeria* species; this ATD (ATG or ATA or ATT) start codon is located 27 bp upstream of the ‘Asn–His–Lys’ site in all *Eimeria* species sequenced to date. The start codon for the COIII CDS was determined to be a TTA codon located 14-20 bp downstream of the LSU/1 (LSUA) region. Use of this conserved start codon produces a COIII product of 252 aa. In all *Eimeria* species studied thus far there is a poly-A- and poly-T-rich region located upstream of both the COI and COIII start codons.

### Phylogenetic analyses

**Figure 2:**
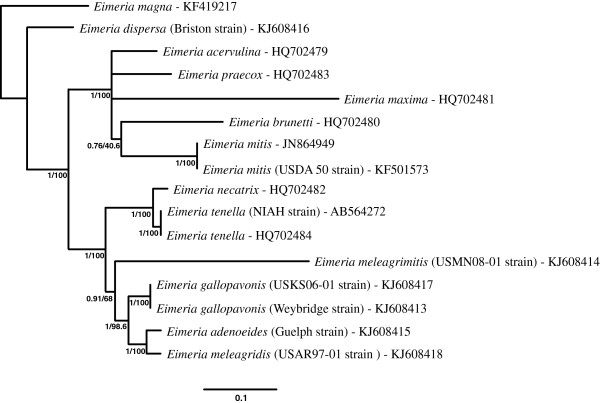
**Bayesian inference and maximum likelihood phylogenetic reconstructions using mitochondrial genome sequences of 16 *Eimeria *species.** The analyses included 5 species infecting turkeys and 7 species infecting chickens and used *Eimeria magna* (a parasite of rabbits) as the functional outgroup to root the tree. Node support is indicated for BI (posterior probability, first number) and for ML (% bootstrap, second number) for all nodes with greater than 0.5 posterior probability. Neither the *Eimeria* species infecting chickens nor the *Eimeria* species infecting turkeys formed monophyletic groups. Both the BI and ML analyses supported monophyly of the 5 *Eimeria* species of chickens that do not usually invade the cecal pouches but branching order among these parasites was poorly resolved in both.

**Figure 3:**
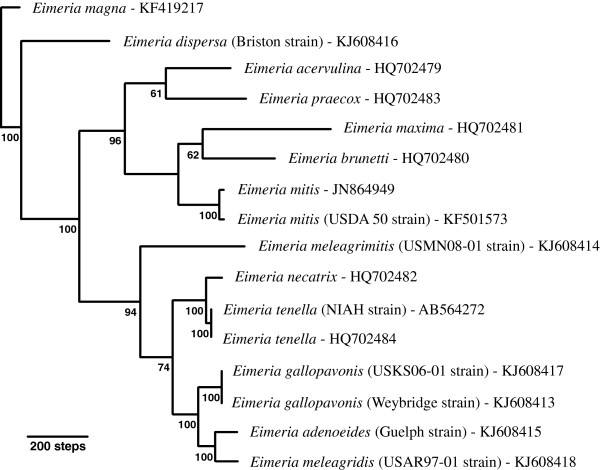
**Maximum parsimony phylogenetic reconstruction using mitochondrial genome sequences of 16 *Eimeria *species.** The analyses included 5 species infecting turkeys and 7 species infecting chickens and used *Eimeria magna* (a parasite of rabbits) as the functional outgroup to root the tree. Tree length was 3147 steps with a consistency index of 0.63 based on 927 parsimony-informative characters; percentage bootstrap support (500 replicates) is indicated at each node. The MP tree differed from the BI/ML tree only in the placement of *E. meleagrimitis* basal to a collection of lower intestinal tract parasites of chickens and turkeys. The MP analysis supported monophyly of the 5 *Eimeria* species of chickens that do not usually invade the cecal pouches.

After trimming the alignment of whole genome sequences to remove the short indel-rich region downstream of the Cocci_WG-MT-R primer, the alignment of 16 available mt whole genome sequences used for phylogenetic analyses was 6416 bp in length, including gaps. The general time reversible model with discrete Gamma (GTR + I + G) distribution of nucleotide substitution [38] was determined to be optimal for the BI and ML analyses. Figure 2 illustrates the phylogenetic relationships based on Bayesian inference (BI) and Maximum likelihood (ML) among the 10 publically available complete mt genome sequences from eimeriid coccidia and the six newly generated complete mt genome sequences from *Eimeria* spp. infecting turkeys. Phylogenetic relationships inferred using a Maximum parsimony (MP) model are illustrated in Figure 3. The *Eimeria magna* mt genome sequence was used as a functional out-group in all phylogenetic analyses. Phylogenetic trees generated from aligned concatenated CDS for COI, COIII and CytB are illustrated in Additional file 1: Figure S1 for BI and ML analyses and Additional file 2: Figure S2 for the MP analysis. Trees generated using concatenated amino acid translations of the CDS matched the trees based on the concatenated CDS dataset under the same phylogenetic inference model (data not shown).

In the BI and ML trees, for global complete mitochondrial nucleotide sequences and the concatenated CDS, all *Eimeria* species causing ‘cecal coccidiosis’ in turkeys (i.e. *E. meleagridis, E. gallopavonis* and *E. adenoeides*) formed a monophyletic clade that was the sister group to *E. meleagrimitis*; the latter species infects the intestinal tract of turkeys excluding the ceca. The *Eimeria* species causing ‘cecal coccidiosis’ in chickens (i.e. *E. tenella* and *E. necatrix*) formed a monophyletic clade that was the sister clade to these four *Eimeria* species infecting turkeys. In the MP trees based on the same DNA sequences (complete genome or concatenated CDS), *Eimeria meleagrimitis* was the sister taxon to a monophyletic clade consisting of species causing ‘cecal coccidiosis’ in chickens and turkeys. In none of the analyses did all *Eimeria* species infecting turkeys form a monophyletic group; in all phylogenetic analyses *E. dispersa* branched near the base of the tree and was the sister taxon to all other *Eimeria* species within the functional ingroup. The *Eimeria* spp infecting chickens, excluding *E. tenella* and *E. necatrix,* formed a monophyletic clade in all analyses and all datasets (DNA and AA-based); however, the branching order within this monophyletic clade varied among analyses. All of these parasites (i.e. *E. acervulina, E. brunetti, E. mitis, E. praecox* and *E. maxima*) infect the intestinal tract of chickens outside of the cecal pouches.

## Discussion

The six newly reported mt genome sequences obtained in this study varied modestly in genome lengths (6165-6238 bp) and were comparable to the lengths (6148 bp to 6408 bp) of the mt genomes of *Eimeria* spp infecting chickens [22-24,39] and rabbits [40]. Genome organization of all mt genome sequences is highly conserved among eimeriid coccidia; however, eimeriid mt genome organization differs markedly from that of other apicomplexan mt genomes e.g. [12,17,20]. No sequence differences (100% sequence identity) were recorded between the two strains of *Eimeria gallopavonis* (i.e. Weybridge strain and USKS06-01 strain) analysed in this study despite being isolated from different geographical regions. The two *E. tenella* sequences isolated from two geographical areas (Japan and China) also did not differ in their sequences [22,23], respectively). In comparison, the two *E. mitis* isolates from the US and China showed sequence differences at 32 positions; perhaps the longer domestication of the chicken host has permitted greater genetic variation in its parasites compared to the domesticated turkey.

The number, direction and lengths of the three CDS were identical in all six mt genome sequences obtained in the present work. Although the COI, COIII and CytB CDS have been annotated inconsistently in the publically available mt genome sequences, alignment of all 16 complete mt genomes from 13 *Eimeria* species demonstrated conserved CDS using the start codons identified by Ogedengbe *et al.*[24] for *E. mitis* USDA50. An assessment of the three CDS across all six genome sequences yielded large numbers of nucleotide substitutions scattered within each gene.

Fragmented rDNA (from 16 to 188 bp in length) annotated in the present study were more highly conserved than the CDS, possibly due to functional constraints in the former. A single rDNA fragment (SSUA (SSU/4) was found upstream of the CytB and COI genes, fifteen rRNA fragments were located between the COI and COIII genes and the ten remaining rRNA fragments were found between the COIII and the end of the mt genome. In addition to rDNA fragments identified in *P. falciparum* that had been previously annotated as putative homologs on *Eimeria* sp. mt genomes see, [24], three regions of each *Eimeria* sp. mt genome had high sequence identities with rDNA fragments encoding RNA14 (SSU/1) or RNA5 (SSU/9) in *P. falciparum* see, [19]. A nearly complete rDNA encoding RNA5 (SSU/9) was located near the 3′ end of each genome and includes the binding site for the Cocci_MT-WG-R primer. The remaining two regions had high sequence identities to two portions of the rDNA encoding RNA14 (SSU/1) in *P. falciparum* see, [19]. However, this rDNA fragment appeared to have been further fragmented on the *Eimeria* sp. mt genomes and the two resulting smaller fragments (29 bp and 41 bp) were found to map to two widely separated regions on these genomes that we annotated as RNA14a (SSU/1a) and RNA14b (SSU/1b), respectively, on all six mt genomes reported in the present study.

All putative ribosomal fragments (fragmented LSU and SSU rDNA) were highly conserved among all *Eimeria* spp [22-24,39,40]; present study. These putative rDNA fragments showed high sequence identity (from 62% to 93.8% pairwise identity) to functionally annotated rRNA fragments of *P. falciparum* M76611 [19]. Occurrence of fragmented and incomplete rRNA genes is not an uncommon phenomenon in apicomplexan parasites; similar fragmented rRNA genes have been reported in all other apicomplexan mt genomes examined to date e.g. [12,17,19,22,23]. Although three additional conserved regions were annotated as putative rDNA fragments in the present study, other highly conserved regions in the six genome sequences remain unannotated but these comparatively conserved regions may represent as yet uncharacterized rDNA fragments.

Phylogenetic analyses under Bayesian, Maximum likelihood and Maximum parsimony evolutionary models using complete mt sequences or concatenated sequences from the three CDS from each mt genome did not support the conclusion that all *Eimeria* species infecting turkeys evolved from a common ancestor. Instead, although many turkey coccidia apparently share a common ancestor, at least one, *E. dispersa* was found branching as the sister taxon to all other *Eimeria* spp in the functional ingroup. It is possible that *E. dispersa* may not have evolved within turkeys but rather arrived in that host via a host switch from some other avian host. *Eimeria dispersa* has been shown to infect both Bobwhite quail (*Colinus virginianus*) and turkeys [41], and perhaps other hosts as well [42]. In addition, the mt genome sequences suggest that the cecal coccidia of chickens (*E. tenella* and *E. necatrix*) are distantly related to the other *Eimeria* of chickens and are more closely related to some of the *Eimeria* spp that infect turkeys; this had been previously suggested on biological [43] and molecular [44] grounds. Analyses of the mt genome sequence data support the suggestion that *Eimeria* spp in chickens represent two distinct ancestral colonisations of the intestine. In one, *E. tenella* and *E. necatrix*, that appear closely related to a number of coccidia infecting turkeys, invaded the ceca of chickens; the remaining five *Eimeria* spp. infecting chickens are closely related using nu 18S rDNA [24,43,44], partial mt COI sequences [24,44] or complete mt genome sequences (current study) and all of these species colonize regions of the intestine excluding the cecum.

Complete mt genome sequences could easily differentiate closely related parasites. For example, the pairwise genetic distance of *E. adenoeides* and *E. meleagridis* of turkeys and *E. tenella* and *E. necatrix* of chickens was 98.2% and 98.4%, respectively. Interestingly, the COI partial sequences for *E. adenoeides* (KCH strain) and *E. adenoeides* (KR strain) [8], are 100% identical to the COI CDS of *E. adenoeides* (Guelph strain) and *E. meleagridis* (USAR97-01 strain), respectively, suggesting that the KCH and KR strains of *E. adenoeides* of Poplstein and Vrba [8] are distinct species rather than strains of a single species.

## Conclusions

The mt genomes of *Eimeria* species infecting turkeys are similar with respect to genome size, organisation, start codon positions and overall base composition with all other *Eimeria* species. Complete mitochondrial genome sequences possess sufficient sequence variability for differentiating *Eimeria* species infecting turkeys or chickens and, in the three cases where more than one complete mt genome is available from a single species (i.e. *E. mitis*, *E. tenella* and *E. gallopavonis*), the intraspecific variation between mt genomes was much smaller (0–0.5%) than the genetic distance between that species and the most closely related *Eimeria* species (1.6 –3.2%). Genetic variability is concentrated within the three CDS encoding COI, COIII and CytB. This makes these mt genes of *Eimeria* spp. suitable (either as individual genes or as concatenated sequences) for species delimitation studies and phylogenetic analyses without the confounding presence of paralogous genome copies encountered with nu rDNA sequences e.g. [24,45]. The nature of the mt genome sequences, and particularly the CDS regions, of *Eimeria* spp. make the mt genome highly suited for development of diagnostic assays as well as, potentially, genetic markers for molecular epidemiology and phylogenetics of coccidia.

## Competing interests

The author(s) declare that they have no competing interests.

## Authors’ contributions

MEO conceived of the study, participated in the design of the study, carried out the molecular genetic studies, DNA extractions and PCR amplifications, participated in the sequence alignment and drafted the manuscript. SES carried out all biological studies including single oocyst isolation from parent stocks providing the parasite material used in the present molecular studies. JW performed parasite isolation, DNA extractions and PCR amplifications. JRB participated in the conception, design and coordination of the study, sequence analyses and helped to draft the manuscript. All authors read and approved the final manuscript.

## Supplementary Material

Additional file 1: Figure S1 Bayesian inference and maximum likelihood phylogenetic reconstructions using mitochondrial CDS sequences of 16 *Eimeria* species. The analyses included 5 species infecting turkeys and 7 species infecting chickens and used *Eimeria magna* (a parasite of rabbits) as the functional outgroup to root the tree. Node support is indicated for BI (posterior probability, first number) and for ML (% bootstrap, second number) for all nodes with greater than 0.5 posterior probability. Neither the *Eimeria* species infecting chickens nor the *Eimeria* species infecting turkeys formed monophyletic groups. Both the BI and ML analyses supported monophyly of the 5 *Eimeria* species of chickens that do not usually invade the cecal pouches but branching order among these parasites was poorly resolved in both. The same tree topology was obtained based on aligned near-complete mitochondrial genome sequences (see Figure 2). Click here for file

Additional file 2: Figure S2 Maximum parsimony phylogenetic reconstruction using mitochondrial CDS sequences of 16 *Eimeria* species. The analyses included 5 species infecting turkeys and 7 species infecting chickens and used *Eimeria magna* (a parasite of rabbits) as the functional outgroup to root the tree. Percentage bootstrap support (500 replicates) is indicated at each node with at least 50% bootstrap support. The MP tree differed from the BI/ML tree only in the placement of *E. meleagrimitis* basal to a collection of lower intestinal tract parasites of chickens and turkeys. The MP analysis supported monophyly of the 5 *Eimeria* species of chickens that do not usually invade the cecal pouches. The same tree topology was obtained based on aligned near-complete mitochondrial genome sequences (see Figure 3). Click here for file

## References

[B1] McDougald LR Saif YM Coccidiosis Diseases of Poultry 2003 Ames, IA, USA: Iowa State Press 974 991

[B2] Chapman H Coccidiosis in the turkey Avian Pathol 2008 37 205 223 18568647 10.1080/03079450802050689

[B3] Dezfoulian O Gharagozlou M Rahbari S Samani R Coccidiosis due to various species of *Eimeria* in the stunted and diarrheic native turkey poults: Pathology and morphological characterization of oocysts Vet Parasitol 2010 65 15 19

[B4] Long PL Millard BJ Shirley MW Strain variation within *Eimeria meleagrimitis* from turkey Parasitology 1977 75 177 182 927886 10.1017/s0031182000062314

[B5] Morrison DA Bornstein S Thebo P Wernery U Kinne J Mattsson JG The current status of the small subunit rRNA phylogeny of the coccidia (Sporozoa) Int J Parasitol 2004 34 501 514 15013740 10.1016/j.ijpara.2003.11.006

[B6] Rampin T Manarolla G Recordati C Sironi G Caecal coccidiosis in commercial male turkeys Ital J Anim Sci 2006 5 315 317

[B7] Cook SM Higuchi DS McGowan AL Schrader JS Withanage GSK Francis MJ Polymerase chain reaction-based identity assay for pathogenic turkey *Eimeria* Avian Dis 2010 54 1152 1156 21313833 10.1637/9271-020310-Reg.1

[B8] Poplstein M Vrba V Description of the two strains of turkey coccidia *Eimeria adenoeides* with remarkable morphological variability Parasitology 2011 138 1211 1216 21810297 10.1017/S0031182011001090

[B9] Ogedengbe JD Hanner RH Barta JR DNA barcoding identifies *Eimeria* species and contributes to the phylogenetics of coccidian parasites (Eimeriorina, Apicomplexa, Alveolata) Int J Parasitol 2011 41 843 850 21515277 10.1016/j.ijpara.2011.03.007

[B10] El-Sherry S Ogedengbe ME Hafeez MA Barta JR Divergent nuclear 18S rDNA paralogs in a turkey coccidium, *Eimeria meleagrimitis*, complicate molecular systematics and identification Int J Parasitol 2013 43 679 685 23639264 10.1016/j.ijpara.2013.03.005

[B11] Feagin JE The extrachromosomal DNAs of apicomplexan parasites Annu Rev Microbiol 1994 48 81 104 7826027 10.1146/annurev.mi.48.100194.000501

[B12] Feagin JE Mitochondrial genome diversity in parasites Int J Parasitol 2000 30 371 390 10731561 10.1016/s0020-7519(99)00190-3

[B13] Gray MW Burger G Lang BF Mitochondrial evolution Science 1999 283 1476 1481 10066161 10.1126/science.283.5407.1476

[B14] Gray MW Burger G Lang BF The origin and early evolution of mitochondria Genome Biol 2001 2 1018.1 1018.5 reviews 10.1186/gb-2001-2-6-reviews1018 PMC138944 11423013

[B15] Preiser PR Wilson RJ Moore PW McCready S Hajibagheri MA Blight KJ Strath M Wiliamson DH Recombination associated with replication of malarial mitochondrial DNA EMBO J 1996 15 684 686 8599952 PMC449987

[B16] He L Zhang Y Zhang QL Zhang WJ Feng HH Khan MK Hu M Zhou YQ Zhao JL Mitochondrial genome of *Babesia orientalis,* apicomplexan parasite of water buffalo (*Bubalus babalis*, Linnaeus, 1758) endemic in China Parasit Vectors 2014 7 82 doi: 10.1186/1756-3305-7-82 24580772 10.1186/1756-3305-7-82 PMC3941609

[B17] Hikosaka K Watanabe Y Tsuji N Kita K Kishine H Arisue N Nirianne MQ Palacpac SK Sawai H Horii T Igarashi I Tanabe K Divergence of the mitochondrial genome structure in the apicomplexan parasites, *Babesia* and *Theileria* Mol Biol Evol 2010 27 1107 1116 20034997 10.1093/molbev/msp320

[B18] Wilson RJ Williamson DH Extrachromosomal DNA in the Apicomplexa Microbiol Mol Biol Rev 1997 61 1 16 9106361 10.1128/mmbr.61.1.1-16.1997 PMC232597

[B19] Feagin JE Harrell MI Lee JC Coe KJ Sands BH Cannone JJ Tami G Schnare MN Gutell RR The Fragmented Mitochondrial Ribosomal RNAs of *Plasmodium falciparum* PLoS One 2012 7 e38320 doi:10.1371/journal.pone.0038320 22761677 10.1371/journal.pone.0038320 PMC3382252

[B20] Gray MW Lang BF Burger G Mitochondria of protists Annu Rev Genet 2004 38 477 524 15568984 10.1146/annurev.genet.37.110801.142526

[B21] Omori S Sato Y Isobe T Yukawa M Murata K Complete nucleotide *s*equences of the mitochondrial genomes of two avian malaria protozoa, *Plasmodium gallinaceum* and *Plasmodium juxtanucleare* Parasitol Res 2007 100 661 664 17047998 10.1007/s00436-006-0333-6

[B22] Hikosaka K Nakai Y Watanabe Y Tachibana S Arisue N Palacpac NM Toyama T Honma H Horii T Kita K Tanabe K Concatenated mitochondrial DNA of the coccidian parasite *Eimeria tenella* Mitochondrion 2011 11 273 278 21047565 10.1016/j.mito.2010.10.003

[B23] Lin RQ Qiu LL Liu GH Wu XY Weng YB Xie WQ Hou J Pan H Yuan ZG Zou FC Hu M Zhu XQ Characterization of the complete mitochondrial genomes of five *Eimeria* species from domestic chickens Gene 2011 408 23 33 10.1016/j.gene.2011.03.004 21402132

[B24] Ogedengbe ME Hafeez AM Barta JR Sequencing the complete mitochondrial genome of *Eimeria mitis* strain USDA50 (Apicomplexa: Eimeriidae) suggests conserved start positions for mtCOI-and mtCOIII-coding regions Parasitol Res 2013 112 4129 4136 24013344 10.1007/s00436-013-3604-z

[B25] El-Sherry S Ogedengbe ME Hafeez MA Sayf-Al-Din M Gad N Barta JR Sequence-based genotyping clarifies conflicting historical morphometric and biological data for five *Eimeria* species infecting turkeys Poult Sciin press 10.3382/ps/peu007 25609693

[B26] El-Sherry S Ogedengbe ME Hafeez MA Sayf-Al-Din M Gad N Barta JR Re-description of a genetically-typed, single oocyst line of the turkey coccidium, *Eimeria adenoeides* Moore and Brown, 1951 Parasitol Resin press 10.1007/s00436-014-4066-7 25127734

[B27] Long PL Millard BJ Studies on *Eimeria dispersa* Tyzzer 1929 in turkeys Parasitology 1979 78 41 52 419003 10.1017/s0031182000048575

[B28] El-Sherry S Rathinam T Hafeez MA Ogedengbe ME Chapman HD Barta JR Biological re-description of a genetically typed, single oocyst line of the turkey coccidium, *Eimeria meleagrimitis* Tyzzer 1929 Parasitol Res 2014 113 1135 1146 24481898 10.1007/s00436-014-3751-x

[B29] Matsler PL Chapman HD Characterization of a strain of *Eimeria meleagridis* from the turkey Avian Dis 2006 50 599 604 17274300 10.1637/7646-051006R.1

[B30] Hein H *Eimeria adenoeides* and *Eimeria meleagrimitis*: Pathogenic effect in turkey poults Exp Parasitol 1969 24 163 170 5777439 10.1016/0014-4894(69)90153-2

[B31] Remmler O McGregor JK A method to facilitate isolation of single coccidial oocysts J Parasitol 1964 50 294 14170769

[B32] Huelsenbeck JP Ronquist F MRBAYES: Bayesian inference of phylogeny Bioinformatics 2001 17 754 755 11524383 10.1093/bioinformatics/17.8.754

[B33] Swofford DL PAUP: Phylogenetic analysis using parsimony (and other methods). Version4 2003 Massachusetts, USA: Sinauer Associates, Sunderland

[B34] Guindon S Dufayard JF Lefort V Anisimova M Hordijk W Gascuel O New algorithms and methods to estimate Maximum-Likelihood phylogenies: Assessing the performance of PhyML 3.0 Syst Biol 2010 59 307 321 20525638 10.1093/sysbio/syq010

[B35] Tamura K Peterson D Peterson N Stecher G Nei M Kumar S MEGA5: Molecular evolutionary genetics analysis using Maximum Likelihood, evolutionary distance, and Maximum Parsimony methods Mol Biol Evol 2011 28 2731 2739 21546353 10.1093/molbev/msr121 PMC3203626

[B36] Ronquist F Huelsenbeck JP MRBAYES 3: Bayesian phylogenetic inference under mixed models Bioinformatics 2003 19 1572 1574 12912839 10.1093/bioinformatics/btg180

[B37] Jones DT Taylor WR Thornton JM The rapid generation of mutation data matrices from protein sequences Comput Appl Biosci 1992 8 275 282 1633570 10.1093/bioinformatics/8.3.275

[B38] Rodriguez F Oliver JL Marin A Medina J The general stochastic model of nucleotide substitution J Theor Biol 1990 142 485 501 2338834 10.1016/s0022-5193(05)80104-3

[B39] Liu GH Hou J Weng YB Song HQ Li S Yuan ZG Lin RQ Zhu X The complete mitochondrial genome sequence of *Eimeria mitis* (Apicomplexa: Coccidia) Mitochondrial DNA 2012 23 341 343 22632170 10.3109/19401736.2012.690750

[B40] Tian SQ Cui P Fang SF Liu GH Wang CR Zhu XQ The complete mitochondrial genome sequence of *Eimeria magna* Mitochondrial DNA 2013 25 in press. doi: 10.3109/19401736.2013.843088 10.3109/19401736.2013.843088 24328820

[B41] Tyzzer EE The coccidia of the pheasant, turkey, and quail Am J Hyg 1929 10 1314 1339

[B42] Yabsley MJ Atkinson CT, Thomas NJ, Hunter DB *Eimeria* Parasitic Diseases of Wild Birds 2009 Ames IA, USA: Wiley –Blackwell 162 180

[B43] Barta JR Martin DS Liberator DA Dashkevicz M Anderson JW Feighner SD Elbrecht A Perkins-Barrow A Jenkins MC Danforth HD Ruff MD Profous-Juchelka H Phylogenetic relationships among eight *Eimeria* species infecting domestic fowl inferred using complete small subunit ribosomal DNA sequences J Parasitol 1997 83 262 271 9105308

[B44] Miska KB Schwarz RS Jenkins MC Rathinam T Chapman HD Molecular characterization and phylogenetic analysis of *Eimeria* from turkeys and game birds: Implications for evolutionary relationships in galliform birds J Parasitol 2010 96 982 986 20950106 10.1645/GE-2344.1

[B45] Vrba V Poplstein M Pakandl M The discovery of the two types of small subunit ribosomal RNA gene in *Eimeria mitis* contests the existence of *E. mivati* as an independent species Vet Parasitol 2011 183 47 53 21767912 10.1016/j.vetpar.2011.06.020

