# Supplementary material for: Complete mitochondrial genome sequences from five Eimeria species (Apicomplexa; Coccidia; Eimeriidae) infecting domestic turkeys
Source: Parasit Vectors. 2014 Jul 17;7:335. doi: 10.1186/1756-3305-7-335 (PMC4223602; doi:10.1186/1756-3305-7-335)
Supplement: Additional file 1: Figure S1 — Bayesian inference and maximum likelihood phylogenetic reconstructions using mitochondrial CDS sequences of 16 Eimeria species. The analyses included 5 species infecting turkeys and 7 species infecting chickens and used Eimeria magna (a parasite of rabbits) as the functional outgroup to root the tree. Node support is indicated for BI (posterior probability, first number) and for ML (% bootstrap, second number) for all nodes with greater than 0.5 posterior probability. Neither the Eimeria species infecting chickens nor the Eimeria species infecting turkeys formed monophyletic groups. Both the BI and ML analyses supported monophyly of the 5 Eimeria species of chickens that do not usually invade the cecal pouches but branching order among these parasites was poorly resolved in both. The same tree topology was obtained based on aligned near-complete mitochondrial genome sequences (see Figure 2). [file 1756-3305-7-335-S1.pptx]

## Slide 1
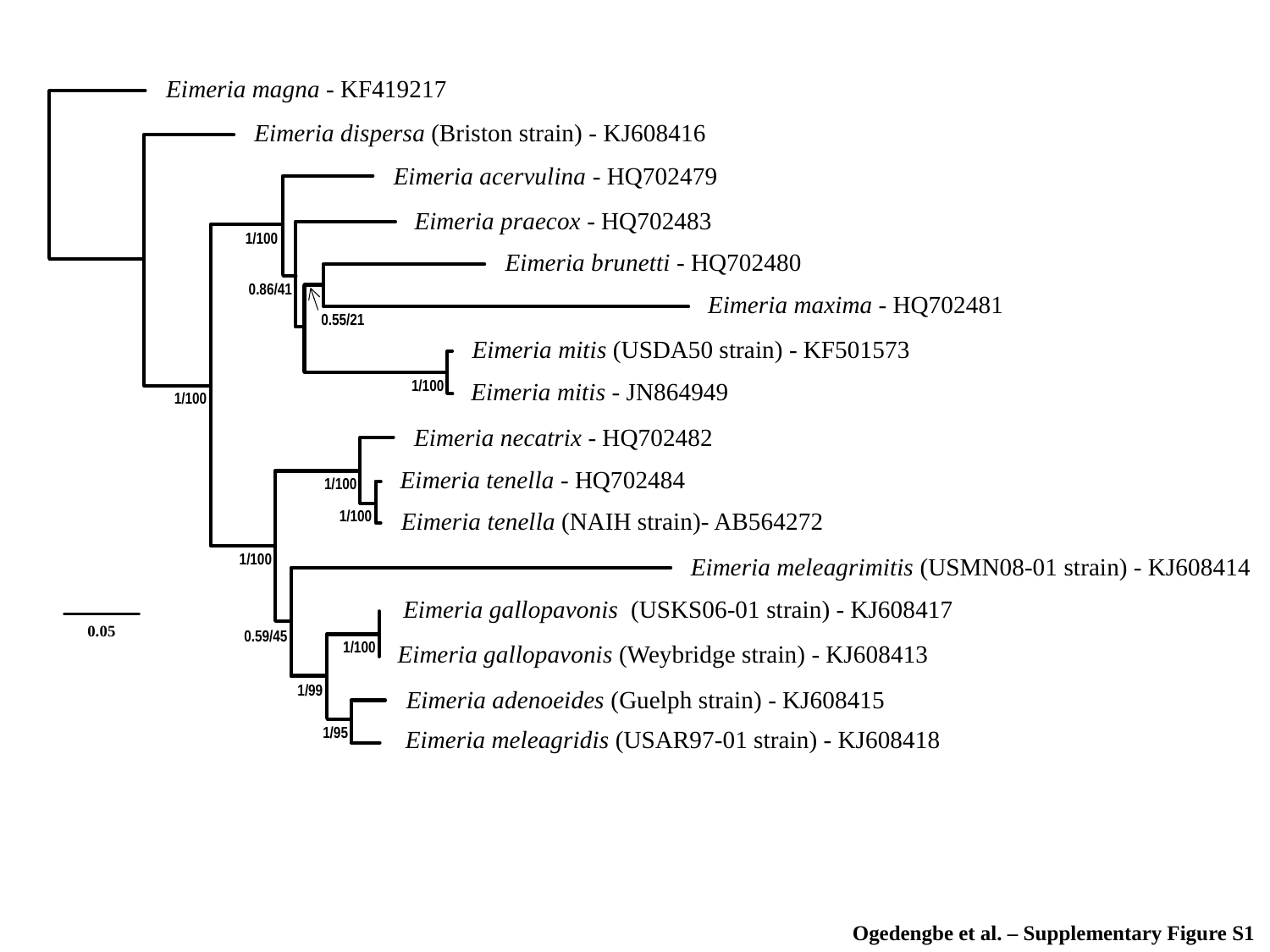

Eimeria magna - KF419217
Eimeria dispersa (Briston strain) - KJ608416
Eimeria acervulina - HQ702479
Eimeria praecox - HQ702483
Eimeria brunetti - HQ702480
Eimeria maxima - HQ702481
Eimeria mitis (USDA50 strain) - KF501573
Eimeria mitis - JN864949
Eimeria necatrix - HQ702482
Eimeria tenella - HQ702484
Eimeria tenella (NAIH strain)- AB564272
Eimeria meleagrimitis (USMN08-01 strain) - KJ608414
Eimeria gallopavonis (USKS06-01 strain) - KJ608417
Eimeria gallopavonis (Weybridge strain) - KJ608413
Eimeria adenoeides (Guelph strain) - KJ608415
Eimeria meleagridis (USAR97-01 strain) - KJ608418
1/100
0.86/41
0.55/21
1/100
1/100
1/100
1/100
1/100
0.59/45
1/100
1/99
1/95
0.05
Ogedengbe et al. – Supplementary Figure S1
