# Supplementary material for: Complete mitochondrial genome sequences from five Eimeria species (Apicomplexa; Coccidia; Eimeriidae) infecting domestic turkeys
Source: Parasit Vectors. 2014 Jul 17;7:335. doi: 10.1186/1756-3305-7-335 (PMC4223602; doi:10.1186/1756-3305-7-335)
Supplement: Additional file 2: Figure S2 — Maximum parsimony phylogenetic reconstruction using mitochondrial CDS sequences of 16 Eimeria species. The analyses included 5 species infecting turkeys and 7 species infecting chickens and used Eimeria magna (a parasite of rabbits) as the functional outgroup to root the tree. Percentage bootstrap support (500 replicates) is indicated at each node with at least 50% bootstrap support. The MP tree differed from the BI/ML tree only in the placement of E. meleagrimitis basal to a collection of lower intestinal tract parasites of chickens and turkeys. The MP analysis supported monophyly of the 5 Eimeria species of chickens that do not usually invade the cecal pouches. The same tree topology was obtained based on aligned near-complete mitochondrial genome sequences (see Figure 3). [file 1756-3305-7-335-S2.pptx]

## Slide 1
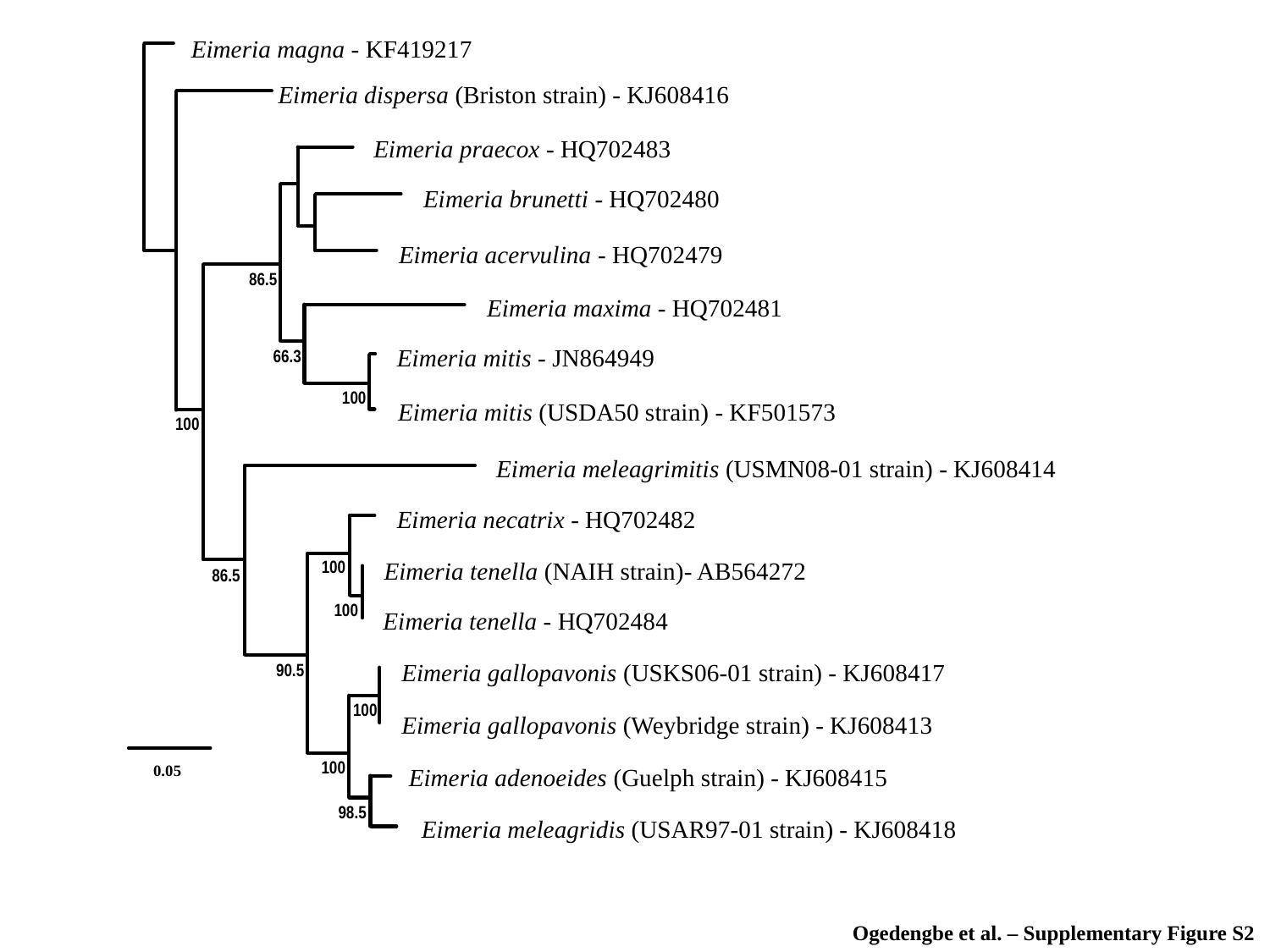

Eimeria magna - KF419217
Eimeria dispersa (Briston strain) - KJ608416
Eimeria praecox - HQ702483
Eimeria brunetti - HQ702480
Eimeria acervulina - HQ702479
Eimeria maxima - HQ702481
Eimeria mitis - JN864949
Eimeria mitis (USDA50 strain) - KF501573
Eimeria meleagrimitis (USMN08-01 strain) - KJ608414
Eimeria necatrix - HQ702482
Eimeria tenella (NAIH strain)- AB564272
Eimeria tenella - HQ702484
Eimeria gallopavonis (USKS06-01 strain) - KJ608417
Eimeria gallopavonis (Weybridge strain) - KJ608413
Eimeria adenoeides (Guelph strain) - KJ608415
Eimeria meleagridis (USAR97-01 strain) - KJ608418
86.5
66.3
100
100
100
86.5
100
90.5
100
100
98.5
0.05
Ogedengbe et al. – Supplementary Figure S2
